# Circular RNA IARS (circ-IARS) secreted by pancreatic cancer cells and located within exosomes regulates endothelial monolayer permeability to promote tumor metastasis

**DOI:** 10.1186/s13046-018-0822-3

**Published:** 2018-07-31

**Authors:** Jie Li, Zhonghu Li, Peng Jiang, Minjie Peng, Xi Zhang, Kai Chen, Hui Liu, Huaqiang Bi, Xiangde Liu, Xiaowu Li

**Affiliations:** 1 Hepatobiliary Surgery Institute, Southwest Hospital, Third Military Medical University (Army Medical University), 30 Gaotanyan Street, Shapingba District, Chongqing, 400038 China; 2 Current address: Hepatobiliary Surgery & Carson International Cancer Shenzhen University General Hospital & Shenzhen University Clinical Medical Academy Center, Shenzhen University, Shenzhen, China

**Keywords:** Exosome, Circular RNA, Endothelial cell, Permeability, Pancreatic cancer, Tumor metastasis

## Abstract

**Background:**

Recent studies show that exosomes are involved in intercellular communication and that abundant circular RNAs (circRNAs) are present within exosomes. However, whether these exosomal circRNAs contribute to tumor invasion and metastasis remains unclear, as do their associated mechanisms.

**Methods:**

Quantitative reverse transcription-polymerase chain reaction (qRT-PCR) was used to measure the expression levels of circ-IARS in 85 pancreatic ductal adenocarcinoma (PDAC) tissues, plasma exosomes, and Hs 766 T, Hs 766 T-L2 and human microvascular vein endothelial (HUVECs) cells. RhoA, ZO-1 and RhoA-GTP levels were detected by qRT-PCR and western blotting (WB); RhoA activity analysis was also performed. Transwell assays were performed to examine changes in endothelial monolayer permeability, and immunofluorescence and WB were employed to evaluate F-actin expression and focal adhesion. Finally, an animal experiment was performed to detect the contribution of circ-IARS to cancer metastasis.

**Results:**

circ-IARS expression was up-regulated in pancreatic cancer tissues and in plasma exosomes of patients with metastatic disease. Circ-IARS was found to enter HUVECs through exosomes and promote tumor invasion and metastasis. Circ-IARS expression was positively correlated with liver metastasis, vascular invasion, and tumor-node-metastasis (TNM) stage and negatively correlated with postoperative survival time. Overexpression of circ-IARS significantly down-regulated miR-122 and ZO-1 levels, up-regulated RhoA and RhoA-GTP levels, increased F-actin expression and focal adhesion, enhanced endothelial monolayer permeability, and promoted tumor invasion and metastasis.

**Conclusions:**

circ-IRAS accesses HUVECs via exosomes derived from pancreatic cancer cells followed by increased endothelial monolayer permeability. Furthermore, this process promotes tumor invasion and metastasis. The results of this study suggest that the presence of circRNAs in exosomes may be important indicator for early diagnosis and prognostic prediction in PDAC.

**Electronic supplementary material:**

The online version of this article (10.1186/s13046-018-0822-3) contains supplementary material, which is available to authorized users.

## Background

Pancreatic ductal adenocarcinoma (PDAC) is a common gastrointestinal cancer that exhibits rapid development, strong invasion and metastasis, and a high mortality rate. Moreover, the incidence rate has shown an upward trend in recent years [1–5]. The high mortality rate of pancreatic cancer is mainly due to difficulties in early diagnosis and lack of knowledge regarding the mechanisms of pancreatic cancer invasion and metastasis [6–8]. Therefore, the discovery of new indicators for early diagnosis and prognostic prediction is critical.

As a type of non-coding RNA (ncRNA) with a closed loop structure and without 5′ and 3′ ends, [9, 10] circular RNAs (circRNAs) are widely found in many organisms and demonstrate obvious tissue specificity [11–15]. Many studies have found that circRNA binds to microRNA (miRNA) via a microRNA response element (MRE) and that circRNA acts as an miRNA sponge, competitively inhibiting miRNA to regulate expression of downstream target genes in post-transcriptional regulation [16, 17]. miR-122 is expressed at low levels in hepatoma and pancreatic cancer, and it may function as a tumor suppressor in these two types of cancer [18, 19]. The endothelium acts as a barrier to control exchange between the blood and surrounding tissues [20, 21]. Increased activity of Ras homolog gene family, member A (RhoA) in human microvascular vein endothelial cells (HUVECs) promotes actin-cytoskeletal remodeling and cell contraction and reduces expression of the tight junction ligand protein Zonula occludens-1 (ZO-1), leading to endothelial barrier dysfunction [22–24] and endothelial hyperpermeability [25–30]. Cancer cells need to cross the endothelial barrier for tumor metastasis, [31] yet research on the involvement of circRNAs in the invasion of blood vessels by tumor cells and the associated mechanisms is scarce.

Exosomes, small vesicles approximately 30–100 nm in diameter [32, 33] with a lipid bilayer membrane structure, [34] are actively secreted by a variety of cells. These vesicles contain proteins, lipids, miRNAs, lncRNAs, and circRNAs [35] and have important functions in the maintenance of physiological status and disease progression [36–39]. Tumor cells constantly release exosomes during tumor development and progression, [40] and this process plays an important role in intercellular communication, which regulates the tumor microenvironment, promotes cell proliferation, and induces cell migration [41]. Exosomes are widely distributed in body fluids and can be taken up by other cells [42] circRNAs are enriched in exosomes [43–45] and may thus be important for intercellular communication [46]. However, studies on exosomal circRNAs and their roles in pancreatic cancer are rare.

In this study, we discovered a new circRNA associated with pancreatic cancer invasion and metastasis. Additional analyses revealed that circ-IARS enters HUVECs through exosomes and that circ-IARS acts as a sponge to absorb miR-122, increases RhoA activity and F-actin expression, reduces ZO-1 expression, enhances endothelial monolayer permeability, and promotes tumor invasion and metastasis. These results provide a new rationale for the investigation of circRNAs in promoting tumor metastasis.

## Methods

### Cell culture

HUVEC, Hs 766 T and Aspc-1 cells were obtained from ATCC. Hs 766 T-L2 cells, the second generation of primary cells from Hs 766 T liver metastatic tissue, were described in our previous report [47]. PDAC cells were cultured in RPMI-1640 medium and HUVECs in Dulbecco’s Modified Eagle’s Medium (DMEM; Gibco, USA) containing 10% fetal bovine serum (FBS) (Zeta-Life, USA) and 1% penicillin-streptomycin solution (Gibco, USA) at 37 °C in a humidified atmosphere containing 5% CO_2_.

### Exosome experiments

Pancreatic cancer cells were cultured with 10% exosome-depleted FBS. Total Exosome Isolation Kit (Thermo, USA) was used according to the manufacturer’s protocol to extract exosomes from 10 ml of culture medium. DiI was added at 2.5 μg/ml to label exosomes, after which the mixture was centrifuged for 1 h at 4 °C. Plasma exosomes were extracted from 500 μl of fresh plasma collected from each patient using Total Exosome Isolation Kit according to the manufacturer’s instructions.

### Transwell analysis

Assays were performed as described previously. HUVECs (6 × 10^4^ at seeding in 200 μl) were cultured for 2 days in a 12-well Transwell chamber with polyester membrane of 8 μm pore size (Corning). To construct an endothelial monolayer model, the medium was changed daily until the cells were completely confluent. HUVECs were stimulated with exosomes (isolated from 10-cm Petri dishes) or phosphate-buffered saline (PBS) as a control for 24 h. For overexpression and interference experiments, 3 × 10^4^ HUVECs were transfected with circ-IARS and miR-122 overexpression or interference plasmid (Sangon Biotech, China) using Lipofectamine 3000 (Invitrogen, USA). Cells cultured until confluence. For transmigration experiments, the medium from the upper chamber was removed, and 3 × 10^4^ GFP-labeled AsPC-1 or Hs 766 T cells were added to 200 μl of serum-free endothelial cell culture medium. In all experiments, tumor cells that passed through the endothelial monolayer and attached to the lower side of the filter were imaged (Olympus) and counted to assess changes in endothelial monolayer permeability. Each experiment was repeated at least three times.

### Microarray analysis

Exosomes from Hs 766 T and Hs 766 T-L2 cells were purified and total RNAs extracted using TRIzol LS (Thermo, USA) according to the manufacturer’s protocol; non-circular RNAs were then removed with RNase R. Arraystar Human circular RNA Array (Arraystar, USA) was used to perform RNA expression profiling. Utilizing a random priming method (Arraystar Super RNA Labeling Kit; Arraystar), circRNAs were amplified and transcribed into fluorescent cDNAs. Arrays were scanned using an Agilent Scanner G2505C, and acquired array images were analyzed with Agilent Feature Extraction software (version 11.0.1.1).

### Transfection

Cell transfections were performed as described previously. For overexpression and interference experiments, 5 × 10^5^ Hs 766 T cells or HUVECs were incubated in a 6-well plate with 2 ml of medium. Plasmid and siRNA transfections were performed using Lipofectamine 3000 with 100 ng DNA or small interfering RNA (siRNA) per well according to the manufacturer’s protocol. siRNA sequences are shown in Additional file 1: Table S1.

### RNA isolation and quantitative reverse transcription-polymerase chain reaction (qRT-PCR) analysis

RNA was isolated using TRIzol LS (Thermo, USA). PrimeScript RT Reagent Kit with gDNA Eraser (TaKaRa, Japan) was used to generate first-strand cDNA; miRNA reverse transcription was carried out using Mir-X miRNA qRT-PCR SYBR Kit (Clontech, Japan). PrimeScript RT Reagent Kit and SYBR Premix Ex Taq (TaKaRa, Japan) were used to perform real-time PCR with a CFX96 Real-Time System (Bio-Rad, USA) and the reaction conditions provided in the instructions. Details of the primers used are presented in Additional file 1: Table S1. The miRNA mRQ 3′ primer was included in Mir-X miRNA qRT-PCR SYBR Kit (Clontech, Japan).

### Western blot analysis

Total protein was extracted from HUVECs using RIPA lysis buffer (Thermo, USA) containing protease inhibitor cocktail tablets (Roche, USA). Protein concentrations were measured with a BCA Protein Assay Kit (Beyotime, China). Equal amounts of protein (20 μg) were separated by 10% sodium dodecyl sulfate polyacrylamide gel electrophoresis (SDS-PAGE) and transferred to polyvinylidene fluoride (PVDF) membranes (Millipore, USA), which were then blocked incubated with primary antibodies overnight at 4 °C. The antibodies used in this study included the following: anti-RhoA (1:600, #2117, Cell Signaling, USA), anti-ZO-1 (1:600, #13663, Cell Signaling, USA) and anti-GAPDH (1:5000, 60,004–1-lg, Proteintech, USA). The membranes were washed with Tris-buffered saline/Tween-20 (TBST) and incubated with a horseradish peroxidase (HRP)-conjugated secondary antibody for 2 h at room temperature. Immunocomplexes were visualized using a New Super ECL Detection Kit (KeyGEN BioTECH, China) according to the manufacturer’s instructions.

### RhoA activity assay

HUVECs were maintained in special medium (with plasma exosomes removed) and cultured at 37 °C for 48 h after the addition of exosomes, plasmids, siRNA, or PBS. After the cell culture medium was removed, the cells were rinsed twice with ice-cold PBS and lysed on ice. RhoA activity detection was conducted according to the instructions of G-LISA RhoA Action Assay Biochem Kit (Kit # BK124, Cytoskeleton, USA). The active form of RhoA, RhoA-GTP, binds to the RhoA-GTP-binding protein immobilized on the bottom of a 96-well plate, whereas the inactive form of RhoA, RhoA-GDP, is removed during washing steps. The bound RhoA-GTP was first incubated with a anti-RhoA for 45 min and then with an HRP-conjugated secondary antibody for 45 min. Absorbance at 490 nm was measured and recorded using a spectrophotometer.

### RhoA-GTP expression analysis

After the cell culture medium was removed, cells were washed twice with ice-cold PBS. The procedures were conducted according to the recommendations of Active Rho Detection Kit (Kit # 8820, Cell Signaling, USA). In this method, the GST-Rhotekin-RBD fusion protein binds to the active form of RhoA (RhoA-GTP) and then immunoprecipitated with glutathione, whereas the inactive form of RhoA (RhoA-GDP) is removed during washing steps. RhoA-GTP expression was detected by western blotting using an anti-RhoA antibody.

### PDAC patients and clinical samples

This study received Ethics Committee of Southwest Hospital approval, and 92 patients signed informed consent. All patients underwent pancreaticoduodenectomy surgery at Department of Hepatobiliary Surgery Institute, Southwest Hospital, from January 2012 to January 2016. Histopathologically confirmed cancer in 92 cases. The clinical characteristics of 85 patients are shown in Table 1. RNA was isolated from 85 fresh frozen tissues and 16 peritumoral normal tissues. After tumor excision, fresh tissues were immediately placed in liquid-nitrogen and then transferred to − 80 °C for future use. Blood was collected from 40 patients for plasma exosome isolation; after centrifugation at 3000 rpm for 20 min, the supernatant (plasma) was stored at − 80 °C for future use. For monthly follow-up, the clinical follow-up center of Department of Hepatobiliary Surgery Institute, Southwest Hospital, telephoned all patients, who were followed up by radiography, ultrasonography or computed tomography (CT) examination every 3 months after discharge.

**Table 1:** The correlation between the clinical characteristics of 85 patients and the expression levels of circ-IARS or linear-IARS

Parameters	circ-IARS			linar-IARS		
	High	Low	*P-value*	High	Low	*P-value*
	(*n* = 42)	(*n* = 43)	/*χ*^*2*^	(*n* = 42)	(*n* = 43)	/*χ*^*2*^
Gender			0.529			0.750
Male	31	35	0.396	32	34	0.101
Female	11	8		10	9	
Age, years			0.235			0.906
≤ 60	20	26	1.412	23	23	0.014
> 60	22	17		19	20	
Tumor location			0.557			0.263
Head	32	35	0.345	31	36	1.250
Body or tail	10	8		11	7	
Tumor size, cm			0.397			0.591
≤ 2	12	16	0.718	15	13	0.289
> 2	30	27		27	30	
Neural invasion			0.595			0.595
Yes	17	15	0.283	17	15	0.283
No	25	28		25	28	
Duodenal invasion			0.715			0.778
Yes	6	5	0.133	5	6	0.079
No	36	38		37	37	
Differentiation			^a^0.410			1.000
Low	11	9	2.119	10	10	0.096
Median	29	28		28	29	
High	2	6		4	4	
Lymphatic invasion			0.058			0.058
Yes	19	11	3.595	19	11	3.595
No	23	32		23	32	
Vascular invasion			**0.020**			0.901
Yes	15	6	5.409	12	9	0.015
No	27	37		30	24	
Liver metastasis			^a^ **0.011**			0.481
Yes	10	2	6.432	8	4	0.496
No	32	41		34	39	
TNM			**0.023**			0.065
I, IIa	18	29	5.195	19	28	3.396
IIb, III and IV	24	14		23	15	

^a^There are at least 1 lattice theoretical frequencies less than 5, so the Fisher exact test is adopted (Monte Carlo test)

Bold value indicates that its *P*-value less than 0.05

### Animal experiment

This study was approved by the Institutional Animal Care and Use Committee of Southwest Hospital, Chongqing, China. Four to six-week-old male athymic nude mice were obtained from Southwest Hospital (Chongqing, China) and raised in the standard pathogen-free conditions of Southwest Hospital (Chongqing, China). Anesthesia was maintained with an intraperitoneal injection of 1% sodium pentobarbital (50 mg/kg). Hs 766 T cells (5 × 10^6^ in 100 μl PBS) were injected into the head of the pancreas after exposing the spleen and pancreas by median abdominal incision. After injection, the pancreas was replaced and the abdomen closed; the mice were imaged using the IVIS Lumina II system (Caliper Life Science, USA). At 4 weeks after the procedure, all mice were sacrificed, and pancreas and liver tissues were fixed in 4% paraformaldehyde and subjected to hematoxylin-eosin (H&E) staining.

### Immunofluorescence

HUVECs were seeded and cultured on cell slides and then transfected with circ-RNA or miRNA or incubated with exosomes. The cells were fixed, permeabilized, and blocked by incubating with 1% bovine serum albumin (Sigma, USA), followed by incubation with anti-Vinculin (1:200, FAK100, Millipore, USA) overnight at 4 °C. The cells were incubated with tetramethylrhodamine (TRITC)-conjugated Phalloidin (1:500, FAK100, Millipore, USA) and a goat anti-mouse IgG (FITC labeled, 1:500, PIERCE, USA) secondary antibody for 1 h at 37 °C. The slides were mounted with Mounting Medium followed Fluorescence with 4′,6-diamidino-2-phenylindole (DAPI) (Vector Laboratories, USA) and imaged with a fluorescence or confocal microscope.

### Statistical analysis

Correlations between clinical categorical parameters and circ-IARS expression levels (the median was regarded as the cutoff value) were evaluated by the χ^2^ test. If the data followed a normal distribution, Student’s t-test was used to compare group differences; otherwise, the nonparametric Mann-Whitney test was adopted. One-way analysis of variance (ANOVA) was employed to compare differences among 3 groups. Univariate analysis was performed by the KM method (the log-rank test), and multivariate analysis was conducted by the stepwise Cox multivariate proportional hazard regression model (Forward LR, likelihood ratio) for survival analysis. All analyses were performed using SPSS 22.0 software (IBM, USA), and all tests were two-sided. A *P*-value < 0.05 was considered to be statistically significant. All statistical analyses were completed under the guidance of experienced experts from the Statistics Department of Third Military Medical University (Army Medical University).

### Dual-luciferase reporter assay

Firstly, 5 × 10^3^ HUVECs were cultured in a white 96-well plate. Then HUVECs were transfected with psiCHECK2-circ-IARS or psiCHECK2-circ-IARS mut plasmid (Sangon Biotech, China) and 8 ng of the internal control pRL-TK Renilla luciferase plasmid (Promega, USA), together with miR-122 (RiboBio, China) at a final concentration of 0, 50 or 150 nM. According to the manufacturer’s protocol, the cells were harvested and processed with the Dual-Luciferase Reporter Assay System (E1910, Promega, USA) after a 48-h incubation. The results were quantified as the ratio of firefly luciferase activity/Renilla luciferase activity in each well.

## Results

### Tumor cell-derived exosomes promote tumor invasion via an increase in endothelial monolayer permeability

**Fig. 1:**
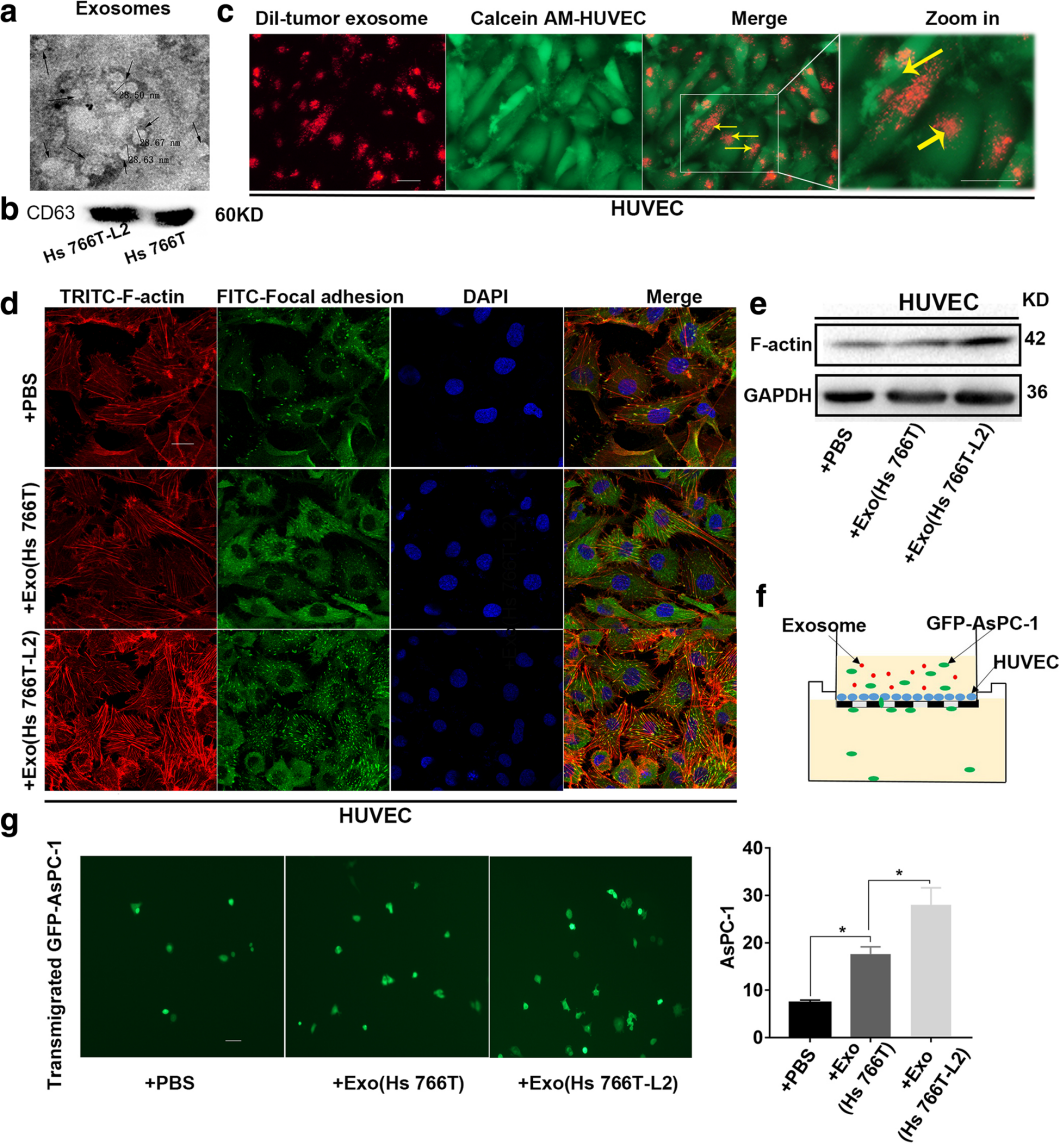
Tumor cell-derived exosomes promote tumor invasion via an increase in endothelial monolayer permeability. **a** Scanning electron microscope was used to examine exosome’s morphology and size. **b** The expression of CD63, in the culture medium of Hs 766 T or Hs 766 T-L2 cells by WB. **c** Exosomes were labeled with DiI dye and co-cultured with Calcein AM-labeled HUVECs for 24 h. Red fluorescent spots were observed in the cytoplasm of HUVECs. Scale bars = 100 μm. **d** HUVECs were seeded and cultured on cell slides and then transfected with exosomes extracted from Hs 766 T or Hs 766 T-L2 cells or PBS as a control. F-actin was labeled with TRITC-conjugated Phalloidin, and focal adhesion sites were labeled with fluorescein isothiocyanate (FITC). The slides were examined by confocal microscopy followed by fluorescence microscopy. Scale bars = 100 μm. **e** WB, which was used to evaluated the expression of F-actin, showed the same results as IF. **f** HUVECs were cultured in a 24-well Transwell upper chamber for 2 days to construct an endothelial monolayer model. **g** Tumor cells that passed through the endothelial monolayer and attached to the lower side of the filter were imaged by microscopy (Olympus) and counted. Scale bars = 50 μm

We collected the culture medium of Hs 766 T cells to isolate exosomes. We extracted the exosomes and examined their morphology and size by scanning electron microscope(SEM)and the expression of CD63, in the culture medium of Hs 766 T or Hs 766 T-L2 cells by WB (Fig. 1a-b). The exosomes were labeled with DiI dye and co-cultured with Calcein AM dye-labeled HUVECs for 24 h. Red fluorescent spots were observed in the HUVECs, indicating that the exosomes had entered these cells (Fig. 1c).

Immunofluorescence (IF) assays showed that compared with the control group, expressions of F-actin and focal adhesion in HUVECs increased significantly after stimulation by exosomes produced by pancreatic cancer cells, with exosomes derived from the Hs 766 T-L2 group showing a more significant increase than those derived from the Hs 766 T group (Fig. 1d); WB, which was used to evaluated the expression of F-actin, showed the same results as IF (Fig. 1e). The number of green fluorescent protein (GFP)-labeled AsPC-1 cells that crossed the endothelial monolayer was greater for the Hs 766 T-L2 and Hs 766 T groups than for the control group, and the number of such GFP-labeled AsPC-1 cells in the Hs 766 T-L2 group was greater than that in the Hs 766 T group (Fig. 1f-g). These results suggest that exosomes secreted by pancreatic cancer cells are taken up by HUVECs, followed by increased F-actin expression, enhanced endothelial monolayer permeability, and tumor cell invasion promotion.

### Circ-IARS is highly expressed in pancreatic cancer, positively correlated with tumor metastasis, and negatively correlated with patient survival time

**Fig. 2:**
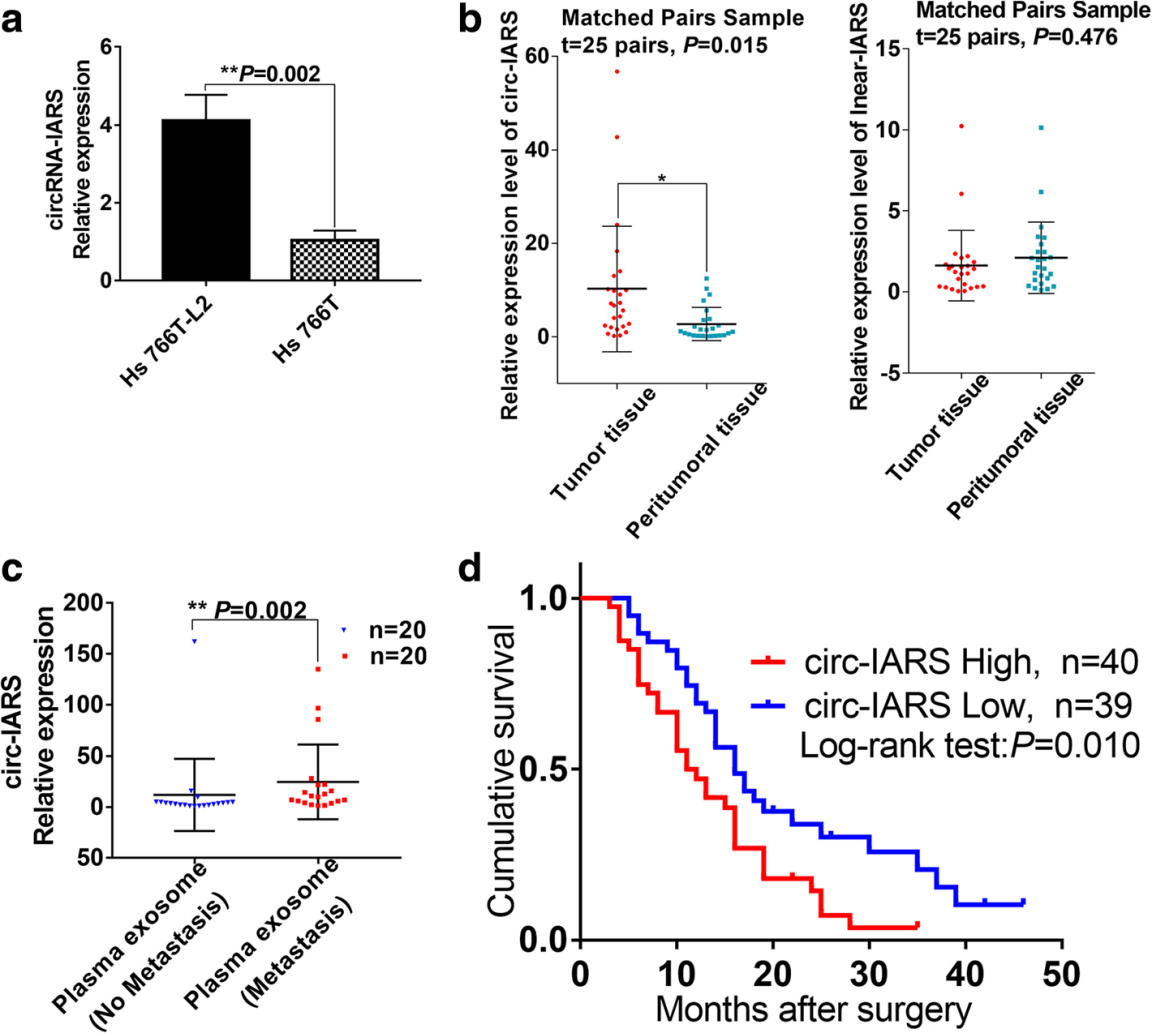
circ-IARS, highly expressed in pancreatic cancer, is an independent risk factor for PDAC patient survival. **a** qRT-PCR validation of the relative expression levels of circ-IARS in Hs 766 T and Hs 766 T-L2 cells. **b** Relative expression levels of circ-IARS and linear-IARS in 25 pairs of pancreatic tumor tissue and adjacent normal tissue. **c** Expression levels of circ-IARS in plasma exosomes from 20 pairs of PDAC patients with or without metastasis were assessed by qRT-PCR. **d** K-M survival curves for survival of all 79 patients with PDAC according to the relative expression levels of circ-IARS in tumor tissues

Using a gene chip, we detected differential expression of circRNAs in exosomes secreted by Hs 766 T-L2 and Hs 766 T cells. Circ-IARS was identified for further investigation via bioinformatic analysis of databases such as circBase/miRBase/TargetScanHuman/microRNA. The results showed circ-IARS to be highly expressed in Hs 766 T-L2 cells (Fig. 2a). This was consistent with the result of the gene chip assay, and we speculate that circ-IARS expression may be related to the invasive capacity of pancreatic cancer cells.

We detected the expression levels of circ-IARS and linear IARS in 25 pairs tumor tissue and peritumoral tissue by qRT-PCR, and found that although circ-IARS was highly expressed in pancreatic cancer tissues, no difference in linear-IARS expression was observed (Fig. 2b). Moreover, circ-IARS was present in abundance in plasma exosomes derived from patients with metastatic pancreatic cancer (Fig. 2c). Statistical analysis showed that high expression levels of circ-IARS were associated with tumor vessel invasion, liver metastasis, and tumor-node-metastasis (TNM) stage; in contrast, linear-IARS was not associated with tumor clinicopathological features (Table 1). Univariate survival analysis showed that vascular invasion, hepatic metastasis, TNM stage, and circ-IARS overexpression were independent risk factors for pancreatic cancer (Table 2). Multivariate survival analysis revealed circ-IARS overexpression, TNM stage, and liver metastasis to be risk factors influencing the prognosis of pancreatic cancer patients (Table 3). circ-IARS survival curves were drawn, and it was found that the survival time of patients in the circ-IARS high-expression group was significantly shorter than that of patients in the circ-IARS low-expression group (Fig. 2d). The above results indicate that expression of circ-IARS is positively correlated with the occurrence and metastasis of pancreatic cancer and negatively correlated with postoperative survival time.

**Table 2:** Univariate analyses of prognostic factors in pancreatic adenocarcinoma

Parameters	Patients, n	Median survial time	*P*-value	*χ* ^*2*^
Gender			**0.016**	5.770
Male	61	16		
Female	18	11		
Age			0.419	0.653
≤ 60	43	14		
> 60	36	14		
Tumor location			0.135	2.233
Head	61	16		
Body, tail	18	11		
Tumor size, cm			0.521	0.412
≤ 2	26	15		
> 2	53	14		
Neural invasion			0.590	0.29
Yes	29	14		
No	50	14		
Duodenal invasion			0.329	0.951
Yes	11	12		
No	68	16		
Differentiation			0.996	0.008
Low	18	14		
Median	54	14		
High	7	18		
Lymphatic invasion			0.202	1.625
Yes	29	11		
No	50	16		
Vascular invasion			**0.038**	4.283
Yes	19	13		
No	60	16		
Liver metastasis			**0.000**	48.761
Yes	12	5		
No	67	16		
TNM			**0.011**	6.400
I, IIa	42	16		
IIb, III and IV	37	11		
circ-IARS			**0.010**	6.633
High	40	11		
Low	39	16		
linar-IARS			0.061	3.517
High	42	13		
Low	37	16		

Bold value indicates that its *P*-value less than 0.05

**Table 3:** Multivariate analyses of prognostic factors in pancreatic adenocarcinoma

Independent factors	Univariate *P*-value	Multivariate *P*-value	Hazard ratio	95% Confidence interval
Gender	0.016	0.108		
Male/Female				
Vascular invasion	0.038	0.509		
Yes/No				
TNM	0.011	**0.037**	1.711	1.033–2.834
I, IIa/IIb, III, IV				
circ-IARS	0.010	**0.033**	1.749	1.047–2.924
High/Low				

Bold value indicates that its *P*-value less than 0.05

### Circ-IARS increases the permeability of endothelial monolayer cells by increasing RhoA activity

**Fig. 3:**
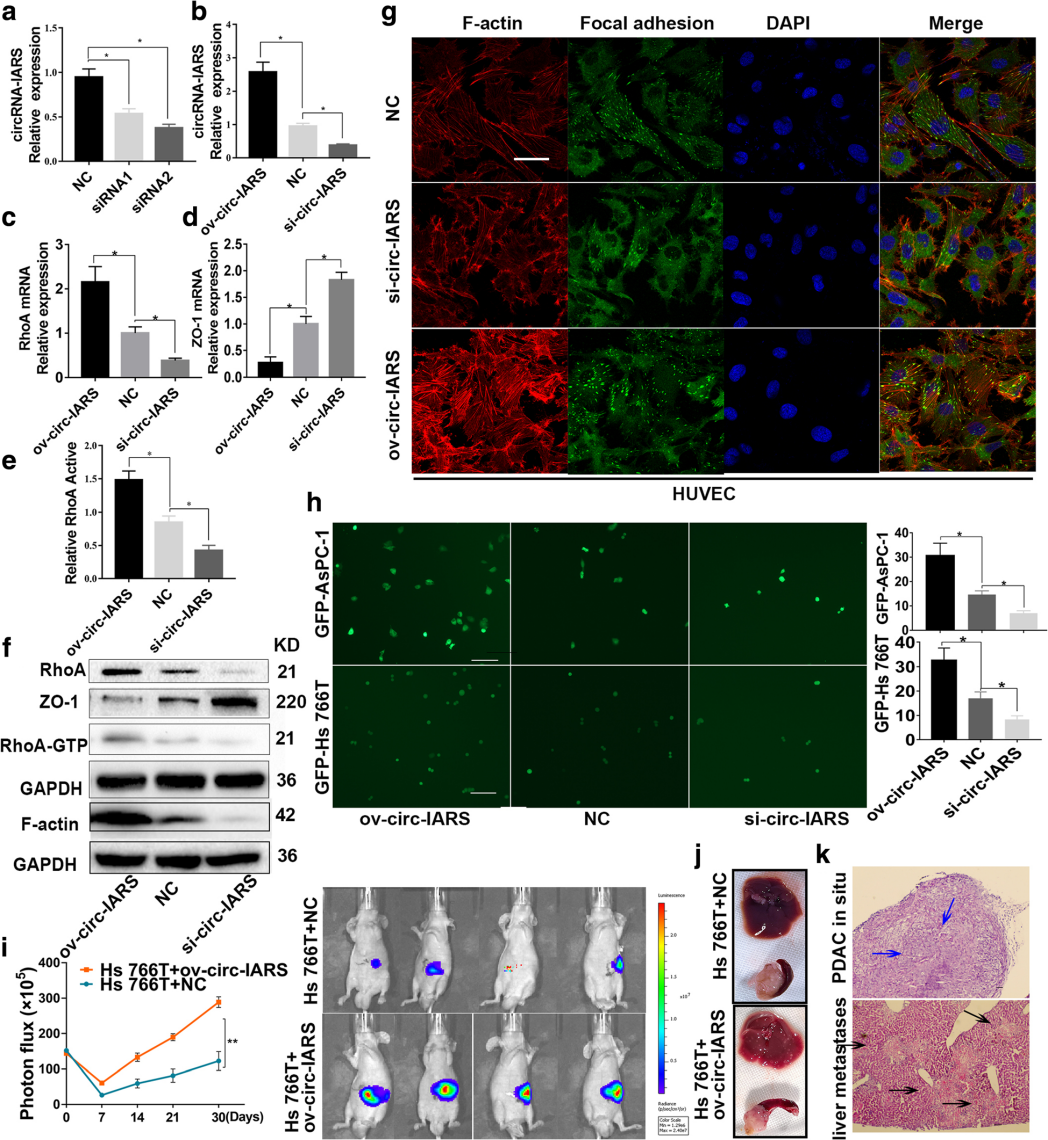
circ-IARS increases the permeability of endothelial monolayer cells by up-regulating RhoA activity. **a** qRT-PCR was used to selected the better interference siRNA, siRNA 2. HUVECs transfected with circ-IARS overexpression plasmids were named the ov-circ-IARS group; HUVECs transfected with circ-IARS siRNAs were named the si-circ-IARS group. **b-d** Relative expression levels of circ-IARS (**b**), RhoA (**c**) and ZO-1(**d**) were measured by qRT-PCR. **e** Relative RhoA activity was significantly increased in the ov-circ-IARS group and decreased in the si-circ-IARS group. **f** Protein levels of RhoA, ZO-1, RhoA-GTP and F-actin were evaluated by western blotting. **g** HUVEC coverslip culture results revealed expression of F-actin and focal adhesion. Scale bars = 100 μm. **h** In a Transwell assay, tumor cells attached to the lower side of the filter were imaged (Olympus) and counted. Scale bars = 50 μm. **i-k** Hs 766 T cells, with circ-IARS up-regulation or NC, were injected into the head of the pancreas in animal experiments to establish a pancreatic cancer tumor model. Images were captured with IVIS Imaging System each week (**i**); a month later, the mice were sacrificed, and the liver or pancreas were checked (**j**). Furthermore, pancreatic cancer in situ or liver metastasis was confirmed by H&E staining; the *blue* arrows indicate pancreatic cancer foci, and the *black* arrows indicate metastatic liver foci. Scale bars = 50 μm (**k**)

In order to down-regulate the expression level of circ-IARS, we designed two siRNAs (Additional file 2: Table S2). Finally, we selected the better interference one, siRNA2, to complete the interference experiment (Fig. 3a). We transfected HUVECs with circ-IARS overexpression plasmids and circ-IARS siRNAs to generate ov-circ-IARS and si-circ-IARS groups, respectively. qRT-PCR showed that the circ-IARS expression level was up-regulated in the ov-circ-IARS group, while down-regulated in si-circ-IARS group (Fig. 3b). In addition, the results of qRT-PCR and western blotting showed that RhoA expression were up-regulated, ZO-1 expression was decreased, and RhoA activity was significantly increased in the ov-circ-IARS group. In contrast, RhoA levels were down-regulated, ZO-1 expression was up-regulated, and RhoA activity was significantly decreased in the si-circ-IARS group (Fig. 3c-f). HUVEC coverslip culture results revealed that circ-IARS overexpression increased F-actin, which was decreased by siRNA-mediated knockdown (Fig. 3g); further, the WB assays revealed the similar results of F-actin in HUVEC cells (Fig. 3f). Transwell assay results showed that the number of AsPC-1 and Hs 66 T cells crossing the epithelial monolayer was significantly increased in the ov-circ-IARS group and significantly decreased in the si-circ-IARS group (Fig. 3h).

To establish a pancreatic cancer tumor model, we constructed a stable circ-IARS-overexpressing cell line, injected it into the head of the pancreas in animal experiments, and periodically monitored the fluorescence signal in this particular region of the pancreas. We found that the signal in the overexpression group was strong and showed a gradually increasing trend (Fig. 3i). After 1 month, larger carcinomas in situ and more liver metastases were found in the circ-IARS overexpression group, i.e., 3 liver metastases in the experimental group and 1 in the control group) (Fig. 3j). We sectioned the pancreatic tumors and liver tissues and conducted H&E staining to confirm the results (Fig. 3k). The above findings indicate that circ-IARS increased RhoA expression and activity as well as F-actin expression and reduced expression of ZO-1, thereby increasing the permeability of endothelial monolayer cells. The animal experiments confirmed that circ-IARS can promote tumor invasion and metastasis in vivo.

### Circ-IARS increases RhoA activity via absorption and regulation of miR122

**Fig. 4:**
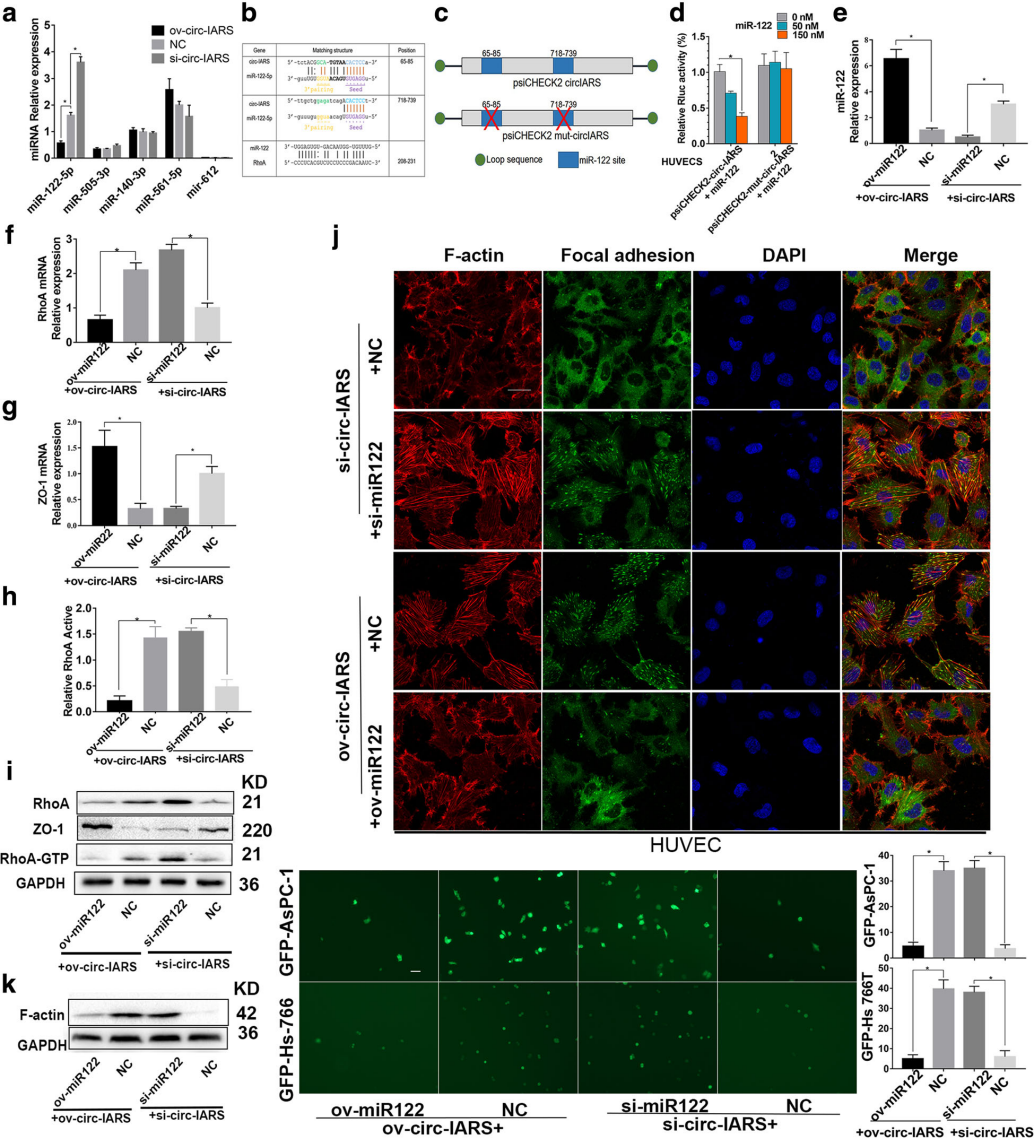
circ-IARS increases RhoA activity via absorption and regulation of miR122. **a** The expression levels of miR-122-5p, miR-140-3p,miR-505-3p, miR-561-5p and miR-612 in circ-IARS-overexpressing and circ-IARS-depleted HUVEC were detected by qRT-PCR. **b** Prediction of miR-122-5P (miR-122) binding sites in circ-IARS and RhoA transcripts. **c-d** The psiCHECK2- circ-IARS plasmid and the psiCHECK2- circ-IARS mut plasmid were constructed to confirm whether circ-IARS acts as a ceRNA to the miR-122. **e** Expression levels of miR-122 in HUVECs up-regulated or down-regulated for circ-IARS or NC. **f-g** qRT-PCR was used to assess relative expression levels of RhoA (**f**) and ZO-1 (**g**) in HUVECs with circ-IARS overexpression or interference or circ-IARS and miR122 together. **h** Relative RhoA activity in HUVECs. **i** Protein levels of RhoA, ZO-1 and RhoA-GTP were measured by western blotting. **j** Expression levels of F-actin and focal adhesion were imaged by confocal microscopy. Scale bars = 100 μm. **k** the changes of F-actin expression in WB were consistent with in IFC. **l** The numbers of AsPC-1 and Hs 766 T cells that passed through the endothelial monolayer were measured in Transwell analysis. Scale bars = 50 μm

In this study, microarray results showed that miR-122-5p, miR-140-3p,miR-505-3p, miR-561-5p and miR-612 maybe regulated by circ-IARS. We then validated the expression levels of these miRNAs in circ-IARS-overexpressing and circ-IARS-depleted HUVEC by qRT-PCR. We found the miR122 was downregulated in circ-IARS-overexpressing cells but upregulated in circ-IARS-depleted cells (Fig. 4a). These results indicated that the miR-122 was the most suitable candidates for further analysis. Bioinformatic analysis showed that circ-IARS or RhoA mRNA bind to a specific site on miRNA122 (Fig. 4b). We have confirmed that circ-IARS can regulate RhoA, but the detailed regulatory mechanism remains unclear. Bioinformatics analysis revealed that circ-IARS shares miR-122 response elements with RhoA, the master molecules of endothelial permeability maintenance. To confirm whether circ-IARS acts as a ceRNA to the miR-122, we constructed the psiCHECK2- circ-IARS plasmid (Fig. 4c). We found that co-transfection of psiCHECK2- circ-IARS and miR-122 inhibited the Rluc expression, and this inhibition was dose dependent, as the inhibitory effect was more obvious in the 150 nM miRNA group than in the 70 nM miRNA group (Fig. 4d, left column). We further constructed the psiCHECK2- circ-IARS mut plasmid. As expected, the mutant no longer elicited the inhibition of miR-122 (Fig. 4d, right column). We confirmed that circ-IARS function as a ceRNA of miR-122 to regulate RhoA expression. Based on qRT-PCR, overexpression of circ-IARS and miR-122 together significantly up-regulated the level of miR-122, decreased that of RhoA, increased that of ZO-1, and decreased RhoA activity compared with the ov-circ-IARS group. In comparison with the si-circ-IARS group, simultaneous interference of circ-IARS and miR-122 significantly down-regulated the level of miR-122, increased expression of RhoA, decreased that of ZO-1, and increased RhoA activity (Fig. 4e-h). The same results were obtained with western blotting (Fig. 4i).

As shown in Fig. 4j, f-actin levels were decreased after overexpression of both circ-IARS and miR-122, whereas F-actin levels were increased after expression of both circ-IARS and miR-122 was simultaneously knocked down; the changes of F-actin expression in WB were consistent with in IF (Fig. 4k). Transwell analysis showed that the numbers of AsPC-1 and Hs 766 T cells that passed through the endothelial monolayer were significantly reduced after circ-IARS and miR-122 were both overexpressed. Moreover, the number of cells that passed through the endothelial monolayer was increased in the si-circ-IARS + si-miR-122 group (Fig. 4l). These results suggest that circ-IARS may increase the permeability of the endothelial monolayer and may promote tumor cell metastasis via specific absorption of miR-122, which reduces its level. This in turn increases the level and activity of RhoA and increases and decreases expression of F-actin and ZO-1, respectively.

### Circ-IRAS enters HUVECs via exosomes and increases endothelial monolayer permeability

**Fig. 5:**
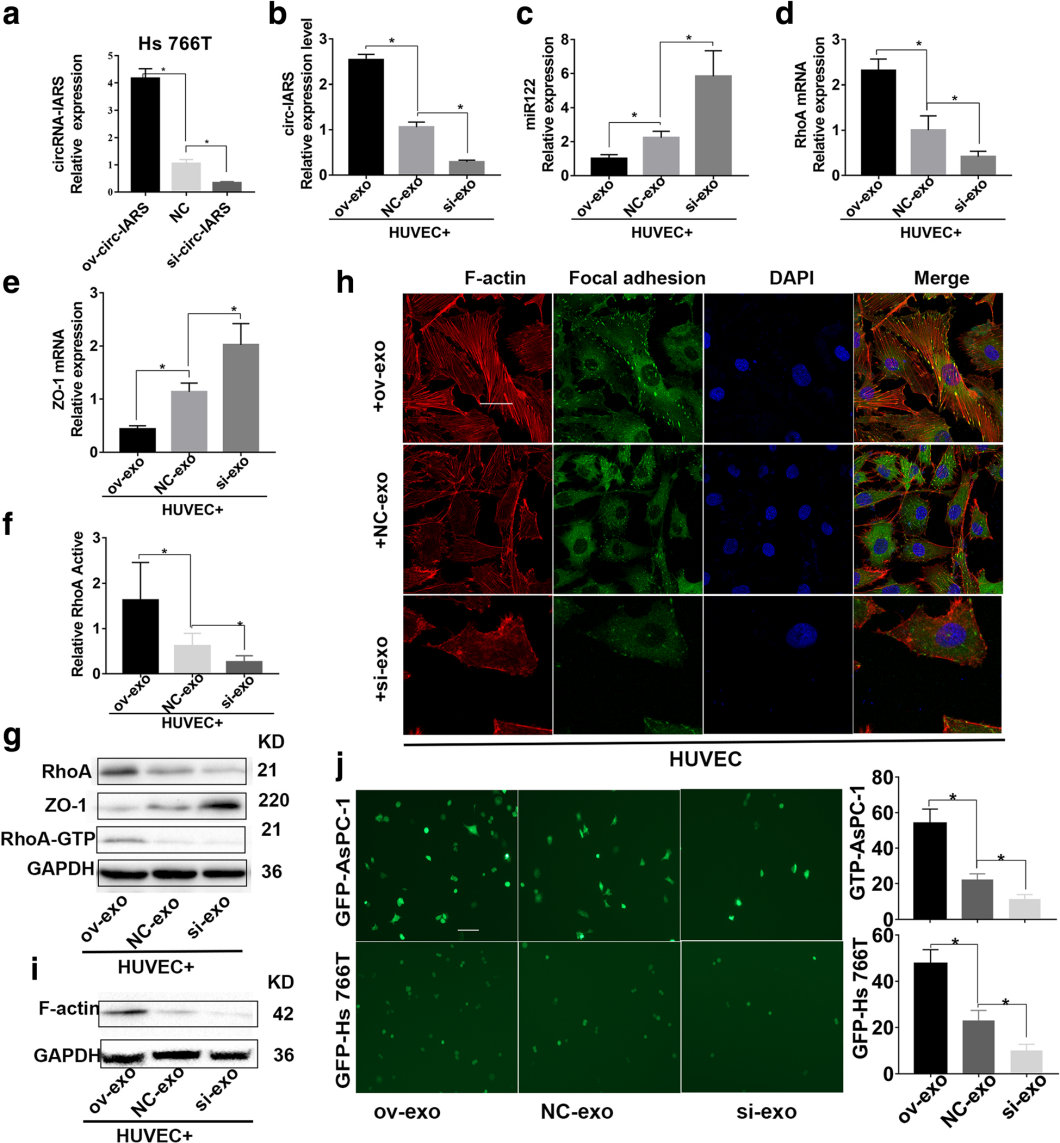
circ-IRAS enters HUVECs via exosomes and increases endothelial monolayer permeability. **a** The expression level of circ-IARS in Hs 766 T was detected by qRT-PCR after transfected with circ-IARS over-expression plasmids or siRNA. Exosomes isolated from Hs 766 T cells transfected with a circ-IARS overexpression plasmid, siRNAs or control were co-cultured with HUVECs. **b-e** Expression levels of circ-IARS (**b**), miR-122 (**c**), RhoA (**d**) and ZO-1 (**e**) were measured by qRT-PCR. **f** Relative RhoA activity was measured in HUVECs. **g** Protein levels of RhoA, ZO-1, RhoA-GTP were analyzed by western blotting. **h** Confocal microscopy was used to image expression of F-actin and focal adhesion. Scale bars = 100 μm. **i** WB found that the change of F-actin is the same as that of IFC. **j** In the Transwell assay, the tumor cells that passed through the endothelial monolayer were imaged by fluorescence microscopy. Scale bars = 50 μm

In our study, the expression level of circ-IARS in Hs 766 T was detected by qRT-PCR after transfection with circ-IARS over-expression plasmids or siRNA. And the expression of circ-IARS was up-regulated in overexpressed Hs 766 T, as the expression was down-regulated in interfered Hs 766 T (Fig. 5a). And then co-cultured the exosomes with HUVECs (HUVEC+ov-exosome and HUVEC+si-exosome groups). The control group (HUVEC+NC-exosome group) consisted of exosomes from untreated Hs 766 T cells. In the HUVEC+ov-exosome group, expression of circ-IARS was significantly increased, that of miR-122 significantly decreased, that of RhoA increased, and that of ZO-1 decreased. Conversely, expression of circ-IARS was significantly down-regulated, that of miR-122 significantly increased, that of RhoA down-regulated, and that of ZO-1 up-regulated in the HUVEC+si-exosome group (Fig. 5b-e). RhoA activity analysis showed increased and decreased activity in the HUVEC+ov-exosome group and HUVEC+si-exosome group, respectively (Fig. 5f). Moreover, the results of western blotting showed that RhoA expression was increased, ZO-1 expression was decreased, and RhoA-GTP expression was increased in the HUVEC+ov-exosome group; however, RhoA and RhoA-GTP expression was decreased and that of ZO-1 increased in the HUVEC+si-exosome group (Fig. 5g).

According to cell coverslip culturing, exosomes overexpressing circ-IARS induced an increase in F-actin expression, whereas exosomes in which circ-IARS was knocked down by siRNA led to decreased F-actin levels (Fig. 5h). In addition, WB found that the change of F-actin is the same as that of IF (Fig. 5i). Transwell assays revealed that exosomes overexpressing circ-IARS increases the number of tumor cells that passed through the endothelial monolayer; in contrast, exosomes of Hs 766 T cells treated with siRNA against circ-IARS decreased the number of tumor cells that passed through the endothelial monolayer (Fig. 5j). The above results indicate that circ-IARS enters HUVECs through exosomes to exert its biological function.

## Discussion

In this study, we investigated the mechanism by which circ-IARS in tumor cell-derived exosomes regulates permeability of the endothelial monolayer and promotes tumor metastasis. The following results are noteworthy. 1. Exosomes secreted by pancreatic cancer cells are taken up by HUVECs, leading to an increase in F-actin expression and enhanced endothelial monolayer permeability; this also promoted the passage of tumor cells through the endothelial monolayer. 2. circ-IARS is strongly expressed in pancreatic cancer, correlating positively with tumor metastasis and negatively with postoperative survival time. 3. circ-IARS increases RhoA expression and activity, increases F-actin levels, decreases ZO-1 expression, and enhances endothelial monolayer permeability. 4. circ-IARS enters HUVECs via exosomes and then exerts its biological function by regulating miR-122 expression.

Previous studies have reported that expression of certain circRNAs, which play an important role in the occurrence and development of tumors, [48, 49] is much higher than that of linear transcripts. [9] Qu et al. examined differential expression of circRNAs in PDAC tissues and normal pancreatic tissues and found that the expression level of circRNA ciRS-7 was negatively correlated with tumor size, stage, and patient clinical prognosis, [50] suggesting that this circRNA may be a new treatment target for pancreatic cancer. We found that circ-IARS was highly expressed in pancreatic cancer tissues and that its expression level was related to tumor vascular invasion, liver metastasis, and TNM stage. Survival analysis showed high circ-IARS expression, TNM stage, and liver metastasis to be risk factors influencing patient prognosis, and the survival curve confirmed that the survival time of the circ-IARS high-expression group was significantly shorter than that of the circ-IARS low-expression group. The endothelium serves as a barrier that controls exchange between the blood and surrounding tissues, and maintenance of this function requires the involvement of active Rho enzyme [51]. An increase in RhoA activity in HUVECs was recently found to increase F-actin expression and reduced that of the tight junction protein ZO-1, [52, 53] which increases the inward contractile force of cells, damages the endothelial barrier function, [22–24] and enhances endothelial permeability [25–30]. After overexpressing circ-IARS in HUVECs, we found that RhoA expression and activity, F-actin expression, and endothelial monolayer permeability were all increased and that ZO-1 expression was decreased. These results suggest that circ-IARS may act through the RhoA signaling pathway to modulate changes in endothelial permeability. Using an animal experiment, we also confirmed that circ-IARS can promote tumor invasion and metastasis. These results suggest that circ-IARS is associated with the progression of pancreatic cancer and may play an important role in this cancer type.

Previous studies indicated that exosomal miR-142-3p augments vascular permeability through down-regulation of endothelial RAB11FIP2 expression [54]. In addition, researchers demonstrated that miR-939 directly targets VE-cadherin leading to an increase in HUVECs monolayer permeability by exosomes [55]. In our study, we found that exosomal circ-IARS could up-regulate the activity and expression of RhoA, increase the expression of F-actin,and decrease the expression of ZO-1. So that, we recognized that High levels of circ-IARS lead to high permeability.

Numerous studies have found that circRNAs can bind to miRNAs as competing endogenous RNAs (ceRNAs) [16, 56, 57] or otherwise directly regulate transcription; [58] alternatively, they may be translated to generate proteins [59–61]. Ashwal-Fluss et al. found that circMbl, which is produced by the second exon of muscleblind (MBL), contains an MBL-binding site that can specifically bind to MBL [62]. In addition, Li et al. found that existing circRNA simultaneously promoted its own expression as well as that of linear RNA, [58] and AbouHaidar MG et al. confirmed that exonic circ RNAs (ecircRNAs) possess an open reading frame that can generate proteins in vivo and in vitro [61]. Other studies have suggested that circRNAs primarily act as miRNA sponges to competitively inhibit miRNAs and to regulate expression of their downstream target genes [16, 17]. Pan et al. found that ciRS-7 acts as an miR-7 sponge by binding to miR-7 and relieves the inhibitory effect of miR-7 on gastric cancer, [63, 64] which suggests that ciRS-7 is an oncogene. Li et al. found that cir-ITCH functions as a sponge that binds to miR-7, miR-17, and miR-214; after the subsequent up-regulation of ITCH and inhibition of the Wnt signaling pathway, it was determined to be a tumor suppressor gene in esophageal cancer [65, 66]. We found that circ-IARS acts as an oncogene to promote tumor cell invasion and metastasis. Wang et al. found that miR-122 can inhibit the invasion and metastasis of hepatocellular carcinoma through the RhoA pathway and that the mechanism involves specific binding of miR-122 to the mRNA sequence of RhoA, [67] inhibiting RhoA activity and F-actin and increasing expression of ZO-1. In our study, we found that expression of miR-122 was decreased by circ-IARS overexpression and increased by interference with circ-IARS expression. Compared with the circ-IARS overexpression group, co-overexpression of circ-IARS and miR-122 resulted in down-regulation of RhoA, increased expression of ZO-1, and a significant decrease in F-actin, as well as decreased endothelial monolayer permeability. In our opinion, circ-IARS binds to miR-122 via sponge-like activity, after which it exerts its biological functions by down-regulating miR-122 expression and relieving its inhibition on the target gene RhoA.

An increasing numbers of studies have found that exosomes play an important role in the occurrence and development of tumors [68, 69]. Kawamoto et al. found that HUVECs employ endocytosis to take up microvesicles secreted by tumors, [70] and we confirmed this finding. We isolated exosomes from the culture medium of tumor cells, labeled them with DiI dye and then co-cultured them with HUVECs, after which we observed the red fluorescent signal carried by the exosomes in HUVECs. Exosomes shuttle information between cells [71] involved in processes such as the immune response, intercellular signal transduction, apoptosis, angiogenesis, and autophagy. Thus, exosomes play an important role in the maintenance of physiological status and disease progression [37–39]. Tumors continuously release exosomes during tumorigenesis and development, at a rate that is much higher than that of normal cells [40]. Wang et al. found that exosomes secreted by prostate cancer cells have an important function in regulating intercellular communication in the tumor microenvironment, thereby promoting cell proliferation and inducing cell migration [41]. In our study, we found that exosomes from pancreatic cancer cells increased F-actin levels in HUVECs (Fig. 1b), enhanced endothelial monolayer permeability, and promoted tumor cell passage through the endothelial monolayer (Fig. 1c). circRNAs are enriched in exosomes and have potential important biological functions. Therefore, to further investigate the role of exosomes in circ-IARS-mediated tumor invasion and metastasis, we co-cultured HUVECs with exosomes derived from Hs 766 T cells with either circ-IARS overexpression or interference. We found that exosomes derived from cells overexpressing circ-IARS led to up-regulation of circ-IARS and down-regulation of miR-122 in HUVECs. Moreover, RhoA expression and activity were significantly increased, expression of ZO-1 was decreased and that of F-actin increased, and endothelial monolayer permeability was enhanced. It is should be addressed that the exogenous circ-IARS, which is secreted from PDAC cells through exosomes, works as the mediators of endothelial monolayer permeability. Uptake of exosomal circular RNA into endothelial cells also suggest that extracellular circular RNAs can be transferred through exosomes from tumor cells to other cells as signaling molecules mediating cell-cell communication.

**Fig. 6:**
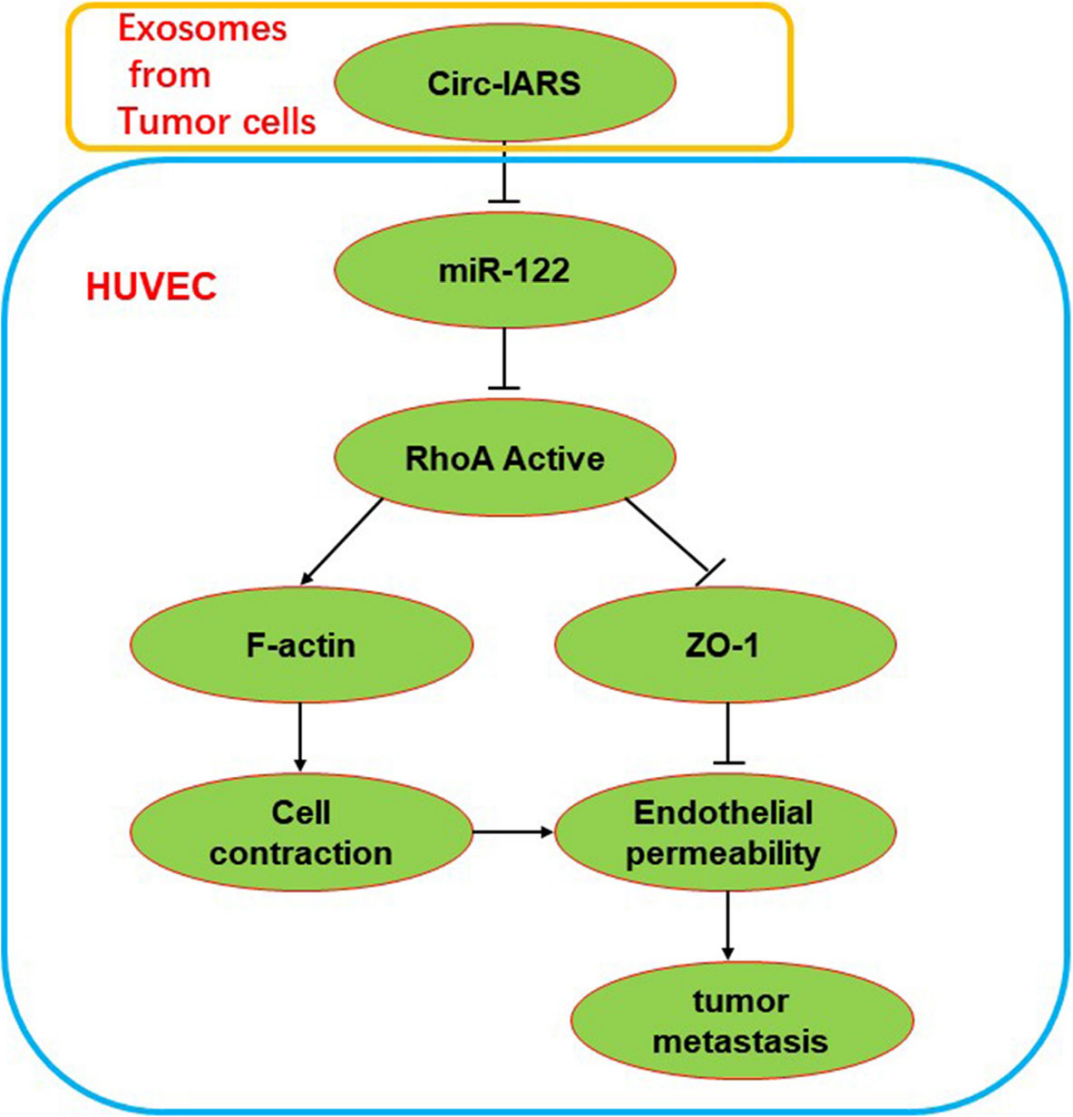
Schematic illustration the relevance among circ-IARS, miR-122, ZO-1, and RhoA expression. Circ-IARS competitively adsorbed miR-122, inhibited its expression and relieved its inhibition of downstream target gene RhoA activity, increased the activity of RhoA, increased the expression of F-actin, and promoted cell contraction. In addition, the increase of RhoA activity reduced the expression of ZO-1 and disrupted the tight junction between the endothelium, which eventually led to the increase of vascular endothelial permeability and promotes tumor metastasis

In conclusion, in this report we describe a novel, physiologically relevant mechanism of vascular permeability regulation. Circ-IARS competitively adsorbed miR-122, inhibited its expression and relieved its inhibition of downstream target gene RhoA activity, increased the activity of RhoA, increased the expression of F-actin, and promoted cell contraction. In addition, the increase of RhoA activity reduced the expression of ZO-1 and disrupted the tight junction between the endothelium, which eventually led to the increase of vascular endothelial permeability and promotes tumor metastasis (Fig. 6). The above results show that tumor cells can regulate endothelial permeability through circ-IARS in exosomes, thus promoting tumor metastasis.

## Conclusions

In summary, we found that exosomes derived from pancreatic cancer cells were taken up by HUVECs and that circ-IARS carried by these exosomes specifically absorbed miR-122 in HUVECs to inhibit its expression and relieve its inhibition of the target gene RhoA. This in turn led to increased RhoA expression levels and activity, thereby further reducing ZO-1 expression and increasing F-actin expression and endothelial monolayer permeability. Overall, this process promotes the passage of tumor cells through the endothelial monolayer as well as tumor invasion and metastasis. We believe that circRNAs in exosomes may be important indicators for the early diagnosis and prognostic prediction of tumors.

## Additional files

Additional file 1: Table S1 Sequences of Primers. (DOCX 19 kb)

Additional file 2: Table S2 siRNA. (DOCX 13 kb)

## Abbreviations

ceRNAs: Competing endogenous RNAs
circ-IARS: Circular RNA IARS
circRNA: Circular RNA
ciRS-7: CircRNA sponge for miR-7
HUVEC: Human microvascular vein endothelial cell
MBL: Muscleblind
MRE: MicroRNA response element
PDAC: Pancreatic ductal adenocarcinoma
qRT-PCR: Quantitative reverse transcription-polymerase chain reaction
RhoA: Ras homolog gene family, member A
RhoA-GTP: the active form of RhoA
siRNA: Small interfering RNA
TNM stage: Tumor-node-metastasis stage
ZO-1: Zonula occludens-1
