# Supplementary material for: Circular RNA IARS (circ-IARS) secreted by pancreatic cancer cells and located within exosomes regulates endothelial monolayer permeability to promote tumor metastasis
Source: J Exp Clin Cancer Res. 2018 Jul 31;37:177. doi: 10.1186/s13046-018-0822-3 (PMC6069563; doi:10.1186/s13046-018-0822-3)
Supplement: Supplementary file 1 — Table S1 Sequences of Primers. (DOCX 19 kb) [file 13046_2018_822_MOESM1_ESM.docx]

| Gene | Primers(5’-3’) |
| --- | --- |
| h-circ-IARS-F | CCAACATTACAGACCGGTG |
| h-circ-IARS-R | CTCGAAGTTGGAAAGTGGAGTG |
| h-linear IARS-F | GGTCAGTGGCGGAACTTGAAGAAC |
| h-linear IARS-R | CAGCGTGAAGGAATGGTCAGGTG |
| h-RhoA-F | AAGAGGCTGGACTCGGATTCGT |
| h-RhoA-R | CCACAGGCTCCATCACCAACAAT |
| h-ZO-1-F | AGGCGGATGGTGCTACAAGTGA |
| h-ZO-1-R | AGAGGACCGTGTAATGGCAGACT |
| h-miR-122-F | GGGTGGAGTGTGACAATGGT |
| h-miR-122-R | CAGTGCGTTCGTGGAGT |
| h-GAPDH-F | AGGGGCCATCCACAGTCTTC |
| h-GAPDH-R | AGAAGGCTGGGGCTCATTTG |
| h-miR-561-5p-F | GCGCGATCAAGGATCTTAAAC |
| h-miR-561-5p-R | CAGTGCGTTCGTGGAGT |
| h-miR-140-3p-F | GCGCGTACCACAGGGTAGAA |
| h-miR-140-3p-R | CAGTGCGTTCGTGGAGT |
| h-miR-505-3p-F | CGCGTCAACACTTGCTGG |
| h-miR-505-3p-R | CAGTGCGTTCGTGGAGT |
| h-mir-612-F | CTGGGCAGGGCTTCTGAG |
| h-mir-612-R | CAGTGCGTTCGTGGAGT |

Table S1. Sequences of Primers
