# Supplementary material for: Circular RNA IARS (circ-IARS) secreted by pancreatic cancer cells and located within exosomes regulates endothelial monolayer permeability to promote tumor metastasis
Source: J Exp Clin Cancer Res. 2018 Jul 31;37:177. doi: 10.1186/s13046-018-0822-3 (PMC6069563; doi:10.1186/s13046-018-0822-3)
Supplement: Supplementary file 2 — Table S2 siRNA. (DOCX 13 kb) [file 13046_2018_822_MOESM2_ESM.docx]

Table s2. siRNA

| circ-IARS siRNA 1 | sense | GAAAUGGCAGGUGGGUCUUTT |
| --- | --- | --- |
|  | antisense | AAGACCCACCUGCCAUUUCTT |
| circ-IARS siRNA 2 | sense | UGGCAGGUGGGUCUUCAAATT |
|  | antisense | UUUGAAGACCCACCUGCCATT |
|  |  |  |
| miR-122 siRNA 1 | sense | AATTCGTGGAGTGTGACAATGGTGTTTGGTTTTGG  CCACTGACTGACCAAACACCTGTCACACTCCACA |
|  | antisense | CCGGTGTGGAGTGTGACAGGTGTTTGGTCAGTCAG  TGGCCAAAACCAAACACCATTGTCACACTCCACG |
| miR-122 siRNA 2 | sense | AATTCCAAACACCATTGTCACACTCCATCA  CCAAACACCATTGTCACACTCCATTTTTTA |
|  | antisense | CCGGTAAAAAATGGAGTGTGACAATGGTGT  TTGGTGATGGAGTGTGACAATGGTGTTTGG |
